# Self management of patients with mild COPD in primary care: randomised controlled trial

**DOI:** 10.1136/bmj.k2241

**Published:** 2018-06-13

**Authors:** Kate Jolly, Manbinder S Sidhu, Catherine A Hewitt, Peter A Coventry, Amanda Daley, Rachel Jordan, Carl Heneghan, Sally Singh, Natalie Ives, Peymane Adab, Susan Jowett, Jinu Varghese, David Nunan, Khaled Ahmed, Lee Dowson, David Fitzmaurice

**Affiliations:** 1 Institute of Applied Health Research, Murray Learning Centre, University of Birmingham, Birmingham, B15 2ER, UK; 2 Department of Health Sciences, University of Leicester, Leicester, UK; 3 Birmingham Clinical Trials Unit, University of Birmingham, Birmingham, UK; 4 Department of Health Sciences, University of York, York, UK; 5 School of Sport, Exercise and Health Sciences, Loughborough University, Loughborough, UK; 6 Nuffield Department of Primary Care Health Sciences, University of Oxford, Oxford, UK; 7 Centre for Exercise and Rehabilitation Science, Biomedical Research Centre (Respiratory), University Hospitals of Leicester NHS Trust, Glenfield Hospital, Leicester UK; 8 Research Institute for Primary Care and Health Sciences, Keele University, Keele, UK; 9 School of Education Research, University of Birmingham, Birmingham, UK; 10 Royal Wolverhampton NHS Trust, New Cross Hospital, Wolverhampton, UK; 11 Warwick Primary Care, University of Warwick, Warwick, UK

## Abstract

**Objective:**

To evaluate the effectiveness of telephone health coaching delivered by a nurse to support self management in a primary care population with mild symptoms of chronic obstructive pulmonary disease (COPD).

**Design:**

Multicentre randomised controlled trial.

**Setting:**

71 general practices in four areas of England.

**Participants:**

577 patients with Medical Research Council dyspnoea scale scores of 1 or 2, recruited from primary care COPD registers with spirometry confirmed diagnosis. Patients were randomised to telephone health coaching (n=289) or usual care (n=288).

**Interventions:**

Telephone health coaching intervention delivered by nurses, underpinned by Social Cognitive Theory. The coaching promoted accessing smoking cessation services, increasing physical activity, medication management, and action planning (4 sessions over 11 weeks; postal information at weeks 16 and 24). The nurses received two days of training. The usual care group received a leaflet about COPD.

**Main outcome measures:**

The primary outcome was health related quality of life at 12 months using the short version of the St George’s Respiratory Questionnaire (SGRQ-C).

**Results:**

The intervention was delivered with good fidelity: 86% of scheduled calls were delivered; 75% of patients received all four calls. 92% of patients were followed-up at six months and 89% at 12 months. There was no difference in SGRQ-C total score at 12 months (mean difference −1.3, 95% confidence interval −3.6 to 0.9, P=0.23). Compared with patients in the usual care group, at six months follow-up, the intervention group reported greater physical activity, more had received a care plan (44% *v* 30%), rescue packs of antibiotics (37% *v* 29%), and inhaler use technique check (68% *v* 55%).

**Conclusions:**

A new telephone health coaching intervention to promote behaviour change in primary care patients with mild symptoms of dyspnoea did lead to changes in self management activities, but did not improve health related quality of life.

**Trial registration:**

Current controlled trials ISRCTN 06710391

## Introduction

Chronic diseases, such as chronic obstructive pulmonary disease (COPD), are a major cause of death and disability in high income countries and of rising importance in low and middle income countries.^1^ Owing to their high prevalence and chronicity, current international policy focuses on the need to support patients to self manage their conditions.^2^ Most interventions designed to support self management have been targeted at people with more severe disease who are likely to be motivated to change behaviour, and where there is the most opportunity for symptomatic improvement. However, more recent efforts have aimed to prevent onset or slow progression early in the disease course to reduce the burden and costs of treating more advanced disease later. This prevention model has only recently been adopted in COPD, with calls for interventions to reduce risk in people with early disease.^3^

The growing number of people at risk of developing long term conditions and the prevalence of early disease, means an accessible and low resource approach needs to be taken to support self management. One such approach is to use interactive telephone health coaching, with the coach and patient working together to identify barriers to behaviour change and finding ways to overcome them. Key techniques include modelling behaviour, goal setting, and empowering the patient to improve their health status.^4^ Telephone health coaching has shown potential benefits on self efficacy, health behaviour, and health status in a rapid review of trials in long term conditions.^5^

COPD is a common respiratory condition with an estimated 65 million people worldwide with moderate or severe disease.^1^ Like most chronic diseases, it causes a considerable burden on health services and society and is a leading cause of death in most countries.^6^ ^7^ Interventions to support self management in patients with COPD have been shown to be effective in improving health related quality of life and in reducing hospital admissions among patients with COPD,^8^ ^9^ but trials have largely recruited people from secondary care and excluded those with mild disease.^9^ However, patients with mild dyspnoea represent 38% to 54% of diagnosed patients in primary care.^10^ ^11^ This is likely to increase with case finding initiatives to identify disease in people with symptoms.^12^

Many components of self management interventions could promote better health and prevent disease progression in the early stages of COPD. Smoking is a major cause of COPD, and smoking cessation has been shown to be beneficial in maintaining better lung function and in slowing disease progression across all severity levels.^13^ ^14^ Reduced physical activity level is an independent risk factor for exacerbations, hospital stays, and mortality among those with COPD and occurs even in the early stages of disease.^15^ ^16^ ^17^ Inhaler treatments have well established efficacy in reducing exacerbations and admissions among patients with moderate and severe COPD, and growing evidence of efficacy in improving clinical outcomes and reducing decline in lung function among patients with more mild impairment.^18^ ^19^ Any intervention that improves medication adherence and inhaler use technique, which is frequently poor,^20^ is thus likely to improve outcomes for patients. Sixty per cent of primary care patients with COPD report exacerbations of their disease,^10^ which are associated with more rapid decline in lung function.^14^ Interventions that aim to reduce the severity of exacerbations include prompting early recognition of symptoms and rapid use of antibiotics or corticosteroids, or both, either through seeking a primary care appointment or use of a self treatment rescue drug pack.

We evaluated telephone health coaching in patients with mildly symptomatic COPD to explore the effectiveness of supporting self management activities in this group of patients. We hypothesised that a telephone health coaching intervention delivered by a nurse to support self management, compared with usual primary care, would lead to improved COPD health related quality of life at 12 months follow-up and would improve physical activity, smoking cessation, self management behaviours, psychological health, and self efficacy.

## Methods

### Design

Patient self management for chronic obstructive pulmonary disease (COPD) was a pragmatic multicentre phase III randomised controlled trial (RCT) of a telephone health coaching intervention to support self management compared with usual care for people with COPD with mild dyspnoea. Details of the study protocol have been published elsewhere.^21^ We followed the CONSORT guidelines for reporting RCTs of non-pharmacological treatments to report this study.^22^ After publication of the protocol in the ISRCTN clinical trial registry at the feasibility study phase, we changed the inclusion criterion for post-bronchodilator spirometry from below the lower limit of normal to forced expiratory volume in one second/forced vital capacity <0.7, which is that recommended by the Global Initiative for Obstructive Lung Disease (GOLD).^6^ We also included some additional subgroup analyses to those in the published protocol (baseline forced expiratory volume in one second predicted (≥80 or <80) and degree of limitation of activities in the St George’s Respiratory Questionnaire (SGRQ-C)).^21^ We embedded a substudy that investigated participant recruitment materials. In this substudy, general practices were randomised to send out either the standard participant information leaflet or a participant information leaflet which contained an additional web address and Quick Response code to give access to web-based materials including podcasts about taking part in research in general and in the patient self management COPD trial in particular.^23^ This did not alter any other trial processes.

### Participants

Participants were recruited from 71 general practices within England located in Birmingham and West Midlands South, Greater Manchester, West Midlands North, and Oxfordshire or Gloucestershire. Patients aged over 18 were identified as eligible if they were on the practice COPD register, thus had respiratory symptoms consistent with COPD; reported mild dyspnoea (MRC grades 1 (only breathless on strenuous exercise) or 2 (only get short of breath when hurrying on level ground or up a slight hill)) at the baseline assessment; had a forced expiratory volume in one second/forced vital capacity <0.7 after post-bronchodilator spirometry (consistent with current UK guidelines)^24^ at the baseline assessment. If there was a contraindication or the patient was unable to perform or refused spirometry, a spirometry result reported from hospital within the last 18 months was used. Doctors were asked to exclude patients who they considered to be inappropriate for the study (eg, had a terminal disease or a severe psychiatric disorder). Eligible patients were sent a letter of invitation, information brochure, and information leaflet from their practice, with a reply slip to the research team which included the MRC dyspnoea scale. Patients with MRC grade 1 or 2, and those without a recorded dyspnoea score were invited to participate.

### Baseline assessment

Patients who expressed an interest in the study were telephoned by a researcher and invited to a recruitment assessment at their practice, undertaken by a research nurse or trained researcher. Patients who attended baseline assessments were given the opportunity to discuss the study. After informed consent, post-bronchodilator spirometry was undertaken, height and weight were measured, and the patient was asked to complete a baseline questionnaire pack. This questionnaire pack included questions on patient demographics and the measures for the primary and secondary outcomes. A GENEActiv accelerometer was fitted on their non-dominant wrist, which they were asked to return by post in a prepaid envelope after seven days of continuous wear. 

### Intervention and usual care

This was a pragmatic trial with no constraints on doctors’ management of the participants in either group.

The usual care group received a standard information leaflet about self management of COPD.^25^ The 13 page leaflet gave a definition of COPD, a detailed description of associated symptoms, how the illness can be managed with the use of inhalers, how to treat exacerbations, and details of other resources (eg, British Lung Foundation and NHS Smokefree).

The intervention consisted of telephone health coaching delivered by a nurse with supporting written documents, a pedometer, and a self monitoring diary. This aimed to support self management in relation to smoking cessation, physical activity increases, correct inhaler use technique, and medication adherence. For those with recurrent exacerbations, it also aimed to improve patient confidence in identifying an exacerbation early in order to start rescue drugs (ie, antibiotics or steroids).

Social Cognitive Theory underpinned the intervention,^26^ and included education, monitoring, and assessment of progress, and taught skills with the aim of increasing self efficacy.^27^ ^28^ We incorporated best evidence for the promotion of physical activity (tailored, ongoing support, duration six months, use of pedometer).^29^ ^30^ ^31^ ^32^ The intervention components are detailed in web appendix 1. The first telephone coaching session at one week after randomisation aimed to last 35-60 minutes (determined by the number of issues requiring discussion, such as current smoking), followed by a 15-20 minute telephone session at weeks 3, 7, and 11 with written supportive materials tailored to the patient after each telephone call (eg, summary of goals agreed, physical activity diary, contact details for local services, information leaflet showing correct inhaler use technique). Nurses provided standard written prompts or information at weeks 16 and 24.

The eight nurses attended two days of training and practiced telephone coaching sessions with the research team. Nurses followed a proforma to guide the consultation in accordance with the telephone consultation protocol. The nurses briefly summarised the content of the call and any actions agreed after each telephone call. A sample of telephone consultations were recorded with the patients consent and reviewed by one researcher to determine compliance with the content of the intervention.

### Randomisation and masking

Patients who had given informed consent and completed all the baseline measures were individually randomised in a 1:1 ratio to the telephone health coaching or usual care group stratified by centre. The allocation was made using a web-based programme hosted by the Primary Care Clinical Research and Trials Unit, University of Birmingham. Centre specific randomisation lists were produced by a statistician at the trials unit. The four recruitment centres were Birmingham and West Midlands South, Greater Manchester, West Midlands North, and Oxfordshire or Gloucestershire. Only the Primary Care Clinical Research and Trials Unit had access to the allocation sequence. Patients were informed of their allocation at the end of the recruitment appointment; they were not masked to treatment allocation. Data were entered into the study database by researchers at the University of Birmingham who were masked to the treatment allocation.

### Outcome assessment

We measured outcomes by postal questionnaire at six months to determine short term change to the end of the intervention and at 12 months to determine whether any change was sustained. At 12 months, accelerometers were posted to participants with a follow-up telephone call to explain how to start the recording. They were asked to wear the accelerometers continuously for seven days and then to return them by post. Non-responders were telephoned and given the option of completing the questionnaire over the telephone.

### Outcomes

The primary outcome measure was health related quality of life at 12 months from randomisation measured using the SGRQ-C.^33^ Scores range from 0 to 100, with higher scores indicating greater impairment of quality of life.

Secondary outcomes were the MRC dyspnoea scale,^34^ self reported physical activity (using the International Physical Activity Questionnaire),^35^ psychological morbidity (using the Hospital Anxiety and Depression Scale),^36^ self efficacy for managing their COPD and undertaking physical activity (using the Stanford self efficacy scale),^28^ and health state utility (using the EuroQoL 5 Dimensions 5 Levels)^37^ at six and 12 months from randomisation. Health related quality of life at six months from randomisation measured using the SGRQ-C was also a secondary outcome. Smoking cessation rates and physical activity measured with GENEActiv accelerometers were assessed at 12 months. Prespecified exploratory outcomes were self management activities (related to smoking cessation, medication adherence, care plans, etc) reported by the patients and healthcare use at six and 12 months. An economic evaluation has also been undertaken, but will be reported elsewhere.

Adverse events were reported by intervention participants during telephone calls and from the six and 12 month follow-up questionnaires; they were independently assessed by two independent clinicians.

### Statistical justification for sample size

The sample size was determined to detect a difference in the SGRQ-C at 12 months. To have 80% power to detect a difference of four points (the minimal clinically significant difference)^38^ from a baseline total score value of 39,^39^ with a standard deviation of 15 at the 5% level of significance required data from 445 patients. Allowing for an attrition rate of 20% at 12 months, we needed 556 participants (278 for each group).

The power to detect differences in self reported physical activity and in smoking cessation rates are detailed in the protocol paper.^21^

### Analysis

All data were analysed by intention to treat. The main analyses compared primary and secondary outcome measures between treatment groups at 12 months after randomisation to assess the long term effect of the self management intervention. Data were also analysed at six months to assess the short term change.

The primary outcome and other continuous secondary outcome measures were analysed using a linear regression model. Ordered categorical secondary outcome measures (eg, MRC dyspnoea scale) were analysed using an ordinal logistic regression model. All primary and secondary analyses were adjusted for baseline values and centre. Differences between treatment groups were summarised using suitable effect estimates (eg, mean difference and odds ratio) with 95% confidence intervals. We used a 5% statistical significance level.

Exploratory outcome measures were not analysed using statistical modelling except for the count data. Binary or categorical outcome measures were analysed using χ^2^ or Fisher’s exact test and continuous measures were analysed using t-tests or a non-parametric equivalent (eg, Wilcoxon rank test). Measures of count were analysed using a Poisson regression model or negative binomial model as appropriate to obtain an incidence rate ratio. Models included an adjustment for baseline values and centre and an offset term for length of follow-up.

Several sensitivity analyses were performed for the SGRQ-C. Firstly, a per-protocol analysis which included only those patients who received all four telephone calls in the intervention group and excluded the one patient in the usual care group who received the intervention by mistake. Secondly, an analysis to assess the effect of missing data, with patients with missing 12 month SGRQ-C scores being simulated by regression imputation using baseline data, with baseline score, age, sex, MRC score, and treatment group used as predictors to impute missing scores. All participants were included in this analysis unless they had died by 12 months or both the baseline and 12 month SGRQ-C scores were missing. Finally, an analysis which excluded participants where the 12 month SGRQ-C questionnaires were returned either early (>1 month before the assessment due date) or late (>65 days after the assessment due date).

Subgroup analyses to explore the effects of the intervention in different patient subgroups were undertaken for the primary outcome. The subgroups, prespecified in the statistical analysis plan included participant characteristics (age, sex, ethnicity, smoking status, baseline MRC dyspnoea score, and number of comorbidities), active engagers with the intervention (through increased physical activity, uptake of smoking cessation support, or checking of inhaler use technique), baseline level of physical activity (from both the International Physical Activity Questionnaire and the accelerometer data), baseline health related quality of life (SGRQ-C), baseline self efficacy (Stanford), and baseline depression and anxiety (from the Hospital Anxiety and Depression Scale). Two post hoc subgroup analyses were also undertaken for baseline forced expiratory volume in one second predicted (≥80 or <80) and degree of limitation of activities in the SGRQ-C. A treatment group by subgroup interaction parameter was included in the linear regression model to assess whether there were any differences in the treatment effect across the different strata. Differences between treatment groups within subgroups were only examined if the interaction parameters were statistically significant (P<0.05).

Details of the accelerometery analyses and available accelerometry data are provided in web appendix 2.

### Patient involvement

The study was supported by a COPD patient advisory group which provided input to a programme of research on COPD. The group met on a regular basis and one was a member of the trial management group for the duration of the study. The group commented on the initial design of the study, the burden of the trial assessment process, participant facing materials, and on the content and material to support the intervention. Additionally, the Trial Steering Group had a lay member. At the end of the study, the group commented on the findings and contributed to the dissemination plan.

## Results

**Fig 1:**
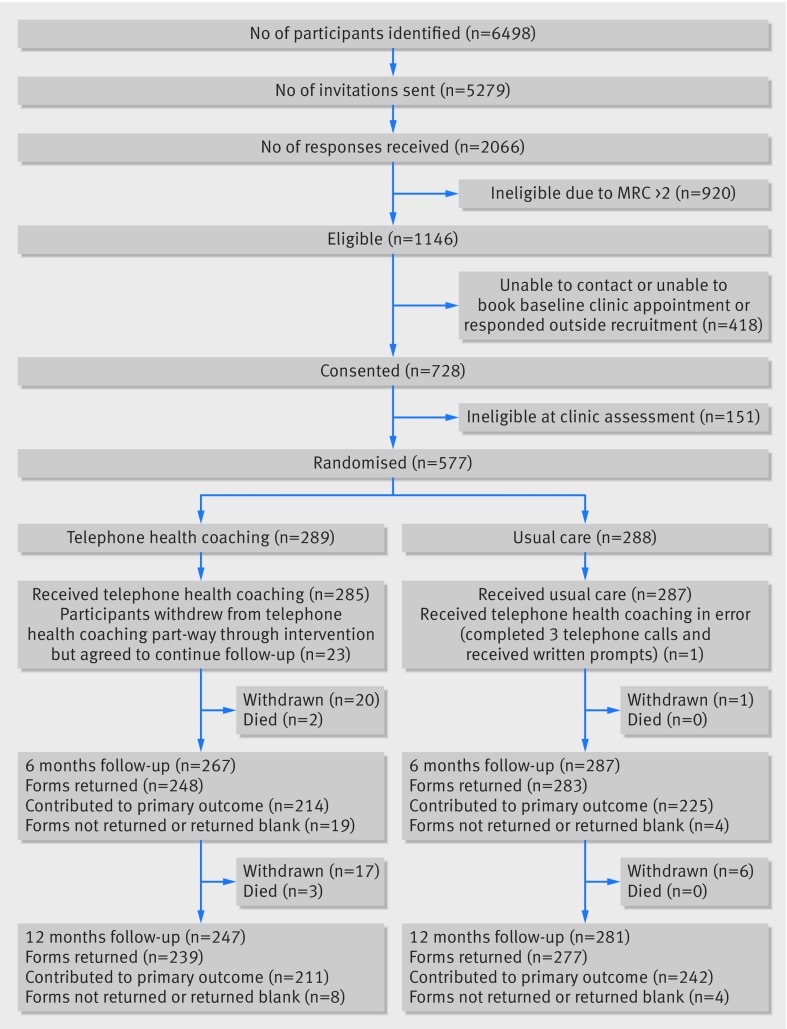
Study flow diagram

We sent a screening questionnaire and invitation leaflet to 5279 people on the COPD registers of 71 general practices; 2066 responded with an interest in the study, but 920 were excluded as they had an MRC dyspnoea scale score of 3 or more. Figure 1 shows that we screened 728 people at their practice between 18 March 2014 and 5 February 2015; 577 were eligible and randomised to telephone health coaching (n=289) or usual care (n=288). In total, 531 (92%) of participants provided data at six months and 516 (89%) at 12 months follow-up. There was imbalance in the follow-up rates between telephone health coaching (82.7%; 37 withdrawals) and usual care (96.2%; 7 withdrawals) groups at 12 months, largely owing to patients who wished to withdraw from telephone health coaching also withdrawing from further follow-up. Of the 37 patients who withdrew from the telephone health coaching group, four withdrew before receiving any intervention and 16 withdrew during the intervention; eight cited illness and 10 cited intervention related factors ranging from it being too demanding to insufficiently so. Seventeen participants withdrew after the six months follow-up. Patients who withdrew from the study did not differ in characteristics to the full sample.

Table 1 shows the patient characteristics. Participants were predominantly white; 63% were male; the mean age was 70.4; 23% were current smokers; and only 19% were in paid employment. Participants had mild disease: mean FEV1 was 71.6% predicted, 193 (33%) were GOLD stage 1 and 309 (54%) GOLD stage 2; 165 (28.6%) reported MRC level I dyspnoea and 270 (47%) reported medication for an exacerbation in the previous 12 months. The mean SGRQ-C total score was 28.7. The study groups were generally well balanced in terms of patient characteristics, although there was a higher proportion of current smokers in the telephone health coaching group. The usual care group reported a higher level of self reported moderate and vigorous physical activity, but this was not reflected in the accelerometry data at baseline. The accelerometry data for all participants showed that participants did a median of 31 minutes of moderate or vigorous activity in bouts of at least 10 minutes daily (interquartile range 0-160). Participants who did not provide data at 12 months were more likely to be in GOLD stage 3, to be smokers, had lower levels of self reported physical activity, and to live alone than responders.

**Table 1:** Baseline patient characteristics. Values are numbers (percentages) unless stated otherwise

Characteristic	Telephone health coaching (n=289)	Usual care (n=288)
Mean (SD) age (years)	70.7 (8.8)	70.2 (7.8)
Age ≥65 years	221 (76)	231 (80)
Men	183 (63)	183 (64)
White race	283 (98)	284 (99)
Median (interquartile range) age at completion of full time education (years)*	15 (15-16)	15 (15-16)
Highest level of qualification:		
No formal qualification	128 (44)	135 (47)
GCSE, CSE, O Level equivalent	58 (20)	63 (22)
A Level, AS Level, or equivalent	27 (9)	24 (8)
Degree level or higher	35 (12)	41 (14)
Other	40 (14)	23 (8)
Missing	1 (<1)	2 (1)
Lives alone	83 (29)	69 (24)
Employment status†:		
Paid work	58 (20)	53 (18)
Unemployed or looking for work	3 (1)	5 (2)
Retired from paid work	216 (75)	214 (74)
Looking after family or home	8 (3)	9 (3)
Unable to work owing to health problems	8 (3)	7 (2)
Other	5 (2)	9 (3)
**Clinical characteristics **		
Current smoker	75 (26)	55 (19)
Mean (SD) body mass index (kg/m^2^)	27.1 (4.4)	27.4 (4.9)
MRC dyspnoea scale score:		
1	89 (31)	76 (26)
2	200 (69)	212 (74)
Mean (SD) FEV1 predicted (%)	71.2 (18.9)	72.1 (18.7)
FEV1 predicted (%):		
<30	1 (<1)	2 (1)
30-49	39 (13)	33 (11)
50-79	160 (55)	149 (52)
≥80	89 (31)	104 (36)
Comorbidities:		
Cancer	34 (12)	37 (13)
Diabetes	32 (11)	36 (13)
High blood pressure	135 (47)	123 (43)
Coronary heart disease	34 (12)	44 (15)
Heart failure	15 (5)	10 (3)
Stroke or mini-stroke	16 (6)	25 (9)
Asthma	98 (34)	100 (35)
Tuberculosis	6 (2)	10 (3)
Osteoarthritis	46 (16)	56 (19)
Rheumatoid arthritis	22 (8)	25 (9)
Osteoporosis	13 (5)	20 (7)
Depression	44 (15)	57 (20)
Other condition	37 (13)	52 (18)
Medication taken regularly for lung problems:		
Beta-2 agonist	201 (70)	197 (68)
Inhaled steroid	27 (9)	39 (14)
Atrovent or Spiriva	109 (38)	117 (41)
Seretide	88 (30)	92 (32)
Symbicort	33 (11)	21 (7)
Theophylline or aminophylline tablets	7 (2)	6 (2)
Steroid tablets	5 (2)	9 (3)
Antibiotic or steroid course, or both, in past 12 months	135 (47)	135 (47)
**Health related quality of life **		
Mean (SD) SGRQ-C total score	27.8 (14.6)	29.5 (14.5)
Mean (SD) SGRQ-C symptoms score	48.5 (21.7)	47.9 (20.7)
Mean (SD) SGRQ-C activity score	36.3 (21.0)	38.7 (21.3)
Mean (SD) SGRQ-C impact score	15.4 (13.4)	17.6 (13.9)
Mean (SD) EuroQoL 5 Dimensions 5 Levels score	0.90 (0.13)	0.89 (0.13)
**Anxiety and depression‡**		
Mean (SD) anxiety subscale score	3.8 (3.4)	4.3 (3.8)
Mean (SD) depression subscale score	2.9 (2.6)	3.1 (2.8)
**Physical activity **		
Mean (SD) minutes of MVPA/week by accelerometry	372.1 (305.1)	379.1 (282.9)
Mean (SD) moderate MET minutes/week§	766.4 (1253.9)	941.5 (1437.6)
Mean (SD) vigorous MET minutes /week§	809.4 (1771.5)	910.2 (1997.4)
**Self efficacy **		
Mean (SD) Stanford self efficacy score	8.3 (1.6)	8.0 (1.7)

FEV1=forced expiratory volume in one second; SGRQ-C=St George's Respiratory Questionnaire; MVPA=moderate to vigorous physical activity; MET=metabolic equivalent.

* One subject in the telephone health coaching group never went to school.

† Not mutually exclusive, participants could tick all that applied.

‡ Using the Hospital Anxiety and Depression Scale.

§ Using the International Physical Activity Questionnaire.

### Intervention delivery

The dose and coverage of intervention delivery was high: 86.4% (999/1156) of the scheduled calls were delivered and 75.4% (218/289) of participants received all four calls. The average duration of calls was 39.2 minutes (SD 10.7) for the first call, then 23.8 (9.2), 21.4 (8.6), and 20.6 minutes (8.7) for the second, third, and final calls respectively. Nurses briefly noted the content and duration of each telephone health coaching session. Most patients spoke to the same nurse for all four calls, although sometimes this was not possible owing to illness or leave. Smoking was discussed in 33% of sessions, physical activity in over 99%, inhaler use technique in 90%, and action planning in 88% of all calls. SMART (specific, measurable, achievable, relevant, and time bound) goals were set in 57% of calls for physical activity, in 11% for smoking cessation, and in 9% for inhaler use technique to be checked.

### Primary outcome

At 12 months, there was no significant difference in the total SGRQ-C score (mean difference −1.3, 95% confidence interval −3.6 to 0.9, P=0.23), although the direction favoured the intervention group. The mean difference in the SGRQ-C activity score was of borderline significance favouring the intervention group (−3.2, −6.3 to 0.0, P=0.05). Table 2 shows that there was no significant difference between groups for the SGRQ-C symptoms or impact scores.

**Table 2:** Comparison of primary and secondary outcomes

Characteristic	Baseline					6 months							12 months					
	Telephone health coaching		Usual care			Telephone health coaching		Usual care		Mean difference (95% CI)	P value		Telephone health coaching		Usual care		Mean difference (95% CI)	P value
	No	Mean (SD)	No	Mean (SD)		No	Mean (SD)	No	Mean (SD)				No	Mean (SD)	No	Mean (SD)		
**Health related quality of life **																		
SGRQ-C total*	277	27.8 (14.6)	272	29.5 (14.5)		222	28.6 (17.1)	237	30.5 (16.7)	−0.3 (−2.3 to 1.7)	0.76		217	27.9 (15.7)	256	30.9 (17.0)	−1.3 (−3.6 to 0.9)	0.23
SGRQ-C symptom*	284	48.5 (21.7)	279	47.9 (20.7)		241	49.5 (22.6)	266	49.2 (21.4)	−0.04 (−2.9 to 2.8)	0.98		230	49.3 (21.4)	273	50.1 (22.6)	−1.9 (−4.9 to 1.1)	0.22
SGRQ-C activity*	281	36.3 (21.0)	279	38.7 (21.3)		229	36.0 (22.7)	252	37.9 (23.9)	0.4 (−2.3 to 3.2)	0.75		224	33.7 (21.1)	260	39.2 (24.4)	−3.2 (−6.3 to 0.0)	0.05
SGRQ-C impact*	286	15.4 (13.4)	280	17.6 (13.9)		233	16.9 (16.3)	255	19.0 (15.5)	−0.7 (−2.8 to 1.4)	0.52		225	16.5 (15.2)	261	19.3 (15.6)	−1.1 (−3.3 to 1.1)	0.33
EQ-5D-5L†	285	0.90 (0.13)	280	0.89 (0.13)		244	0.88 (0.16)	272	0.87 (0.14)	0.01 (−0.01 to 0.03)	0.30		235	0.87 (0.14)	270	0.86 (0.17)	0.01 (−0.01 to 0.03)	0.38
**Anxiety and depression **																		
HADS anxiety subscale score*	286	3.8 (3.4)	285	4.3 (3.8)		243	3.8 (3.8)	279	4.5 (4.0)	−0.3 (−0.8 to 0.2)	0.21		227	4.0 (3.8)	267	4.7 (4.0)	−0.06 (−0.6 to 0.4)	0.81
HADS depression subscale score*	287	2.9 (2.6)	285	3.1 (2.8)		244	3.1 (3.0)	279	3.5 (3.1)	−0.2 (−0.6 to 0.1)	0.21		228	3.3 (3.3)	270	3.8 (3.4)	−0.1 (−0.6 to 0.4)	0.63
**Self efficacy **																		
Stanford self efficacy score†	287	8.3 (1.6)	284	8.0 (1.7)		247	8.1 (1.7)	275	7.8 (1.8)	0.2 (−0.07 to 0.4)	0.16		228	8.1 (1.6)	272	7.7 (1.8)	0.1 (−0.1 to 0.4)	0.32
**Physical activity (accelerometry) **																		
MVPA minutes/week†	263	372.1 (305.1)	259	379.1 (282.9)		NA	NA	NA	NA	NA	NA		179	346.5 (276.6)	232	315.5 (256.1)	11.8 (−21.1 to 44.8)	0.48
**Physical activity (International Physical Activity Questionnaire) **																		
Total MET minutes/week†	230	3242.2 (3284.2)	236	3265.8 (3480.6)		202	3786.0 (3685.7)	237	2920.6 (3195.0)	924.7 (318.3 to 1531.1)	0.003		191	3214.3 (3578.4)	223	2738.1 (3249.9)	410.0 (−235.7 to 1055.7)	0.21
Walking MET minutes/week†	249	1496.0 (1324.6)	253	1371.0 (1249.0)		216	1728.8 (1390.6)	248	1404.2 (1244.0)	283.4 (55.2 to 511.6)	0.02		200	1588.5 (1386.7)	234	1362.7 (1318.2)	161.5 (−86.2 to 409.3)	0.20
Moderate MET minutes/week†	267	766.4 (1253.9)	265	941.5 (1437.6)		236	950.8 (1399.7)	268	732.9 (1208.2)	233.9 (10.6 to 457.1)	0.04		218	765.9 (1256.4)	261	628.6 (1164.8)	130.1 (−83.9 to 344.0)	0.23
Vigorous MET minutes /week†	271	809.4 (1771.5)	282	910.2 (1997.4)		229	1050.5 (2212.7)	270	728.7 (1656.6)	335.8 (23.2 to 648.4)	0.04		218	864.4 (1994.3)	264	705.3 (1674.0)	160.1 (−141.1 to 461.3)	0.30

SGRQ-C=St George's Respiratory Questionnaire; EQ-5D-5L=EuroQoL 5 Dimensions 5 Levels; HADS=Hospital Anxiety and Depression Scale; MVPA=moderate to vigorous physical activity; NA=not available; MET=metabolic equivalent.

* Telephone health coaching compared with usual care (negative values favour telephone health coaching).

† Telephone health coaching compared with usual care (positive values favour telephone health coaching).

### Secondary outcomes

Table 2 shows that at six months, there were no significant differences in the SGRQ-C total and subscores. At six and 12 months, there were also no differences in the EuroQoL 5 Dimensions 5 Levels, Hospital Anxiety and Depression Scale, Stanford self efficacy scale, or level of breathlessness (MRC, table 3). At six months, total self reported physical activity, walking, moderate, and vigorous intensity activity were all significantly higher in the intervention arm (table 2). Differences favoured the intervention arm at 12 months, but they did not remain statistically significant. There was no difference in moderate or vigorous activity measured using accelerometry at 12 months. There was also no difference in smoking cessation rates at six and 12 months (table 3).

**Table 3:** Comparison of secondary outcomes: breathlessness and smoking. Values are numbers (percentages) unless stated otherwise

Characteristic	Baseline			6 months					12 months			
	Telephone health coaching	Usual care		Telephone health coaching	Usual care	Odds ratio (95% CI)*	P value		Telephone health coaching	Usual care	Odds ratio (95% CI)*	P value
MRC Dyspnoea scale score:						0.8 (0.6 to 1.2)	0.39				1.1 (0.7 to 1.5)	0.79
1	89 (31)	76 (26)		77 (32)	84 (32)				69 (31)	74 (28)		
2	200 (69)	212 (74)		137 (58)	158 (60)				137 (61)	163 (61)		
3	NA	NA		14 (6)	18 (7)				16 (7)	27 (10)		
4	NA	NA		8 (3)	5 (2)				4 (2)	5 (2)		
5	NA	NA		1 (<1)	0 (NA)				0 (NA)	0 (NA)		
Quit smoking†	75 (26)	55 (19)		14 (22)	9 (18)	NA	0.60		7 (13)	13 (25)	NA	0.11

NA=not applicable.

* Telephone health coaching compared with usual care (odds ratio >1 favours telephone health coaching).

† Baseline data are reported as current smokers at baseline. Only patients who reported they were current smokers at baseline were included in the analyses on quitting smoking at 6 and 12 months.

### Healthcare utilisation

At six months, intervention participants reported lower doctor and pharmacist consultations, but higher all cause emergency department visits. There were no differences at 12 months (table 4). At six and 12 months, 106 (43%) and 89 (37%) of the intervention group respectively had been prescribed at least one course of antibiotics compared with 105 (37%) and 96 (35%) of the usual care group (data not shown).

**Table 4:** Healthcare utilisation

Characteristic	Baseline					6 months							12 months					
	Telephone health coaching		Usual care			Telephone health coaching		Usual care		Incidence rate ratio* (95% CI)	P value†		Telephone health coaching		Usual care		Incidence rate ratio* (95% CI)	P value†
	No	Mean (SD)	No	Mean (SD)		No	Mean (SD)	No	Mean (SD)				No	Mean (SD)	No	Mean (SD)		
**Hospital admissions**																		
All cause	277	0.11 (0.4)	281	0.12 (0.4)		248	0.07 (0.3)	283	0.08 (0.3)	0.86 (0.45 to 1.62)	0.64		239	0.06 (0.3)	277	0.06 (0.3)	0.90‡ (0.39 to 2.09)	0.81
Respiratory	281	0.02 (0.2)	283	0.02 (0.1)		248	0.02 (0.1)	283	0.03 (0.2)	0.55‡ (0.15 to 2.07)	0.38		239	0.01 (0.1)	277	0.01 (0.1)	0.56‡ (0.08 to 4.11)	0.57
**Emergency department**																		
All cause	286	0.22 (0.5)	280	0.23 (0.6)		248	0.33 (1.8)	283	0.16 (0.5)	1.87‡ (1.06 to 3.27)	0.03		238	0.26 (0.8)	276	0.23 (0.7)	1.06‡ (0.62 to 1.83)	0.82
Respiratory	286	0.04 (0.2)	282	0.02 (0.1)		248	0.04 (0.2)	283	0.03 (0.2)	1.48‡ (0.50 to 4.43)	0.48		239	0.04 (0.2)	277	0.01 (0.1)	2.85‡ (0.67 to 12.20)	0.16
**Primary care consultations**																		
All cause:																		
Doctor	288	1.18 (1.7)	285	1.23 (1.5)		243	1.41 (1.5)	282	1.76 (2.2)	0.80‡ (0.66 to 0.96)	0.02		236	1.45 (1.8)	272	1.67 (1.8)	0.85‡ (0.70 to 1.03)	0.09
Practice nurse	288	0.60 (1.8)	285	0.65 (1.4)		243	0.71 (1.5)	282	0.72 (1.4)	0.98‡ (0.73 to 1.31)	0.88		234	0.56 (1.0)	272	0.67 (1.0)	0.81‡ (0.62 to 1.07)	0.14
Pharmacist	288	0.10 (0.9)	285	0.13 (0.6)		243	0.05 (0.4)	282	0.17 (0.8)	0.27‡ (0.10 to 0.75)	0.01		233	0.10 (0.6)	268	0.11 (0.5)	0.75‡ (0.29 to 1.91)	0.54
Respiratory	186	0.63 (0.9)	190	0.84 (1.1)		164	0.90 (1.1)	197	0.96 (1.3)	0.99‡ (0.72 to 1.35)	0.93		132	0.77 (1.0)	167	0.83 (1.2)	0.84‡ (0.60 to 1.19)	0.34

* Telephone health coaching compared with usual care (Incidence Rate Ratio <1 favours telephone health coaching).

† Statistical significance determined from a χ^2^ test.

‡ Estimate from a negative binomial model rather than a Poisson regression model.

### Activities targeted by telephone health coaching

Physical activity and smoking cessation rates have been described previously. There was no difference between the groups in smoking quit attempts in the previous six months or attendance at smoking cessation services at either follow-up point. At six months, participants in the intervention group reported improved medication adherence, with significantly higher proportions having an inhaler check in the past six months (68% *v* 55%), an agreed care plan with a healthcare provider (44% *v* 30%), written advice about what to do if symptoms worsened (23% *v* 17%), and having an antibiotic rescue pack (37% *v* 29%). However, they did not report improved confidence in the use of rescue packs. Table 5 shows that many of these improvements were sustained at 12 months.

**Table 5:** Self reported self management behaviours. Value are numbers (percentages) unless stated otherwise

Variables	Baseline			6 months				12 months		
	Telephone health coaching	Usual care		Telephone health coaching	Usual care	P value		Telephone health coaching	Usual care	P value
Smoking quit attempt in past 6 months	NA	NA		29 (54)	21 (49)	0.72		30 (58)	21 (53)	0.55
Attendance at smoking cessation service*	NA	NA		3 (10)	1 (5)	0.63		3 (10)	4 (19)	0.43
Median (IQR, n) medication adherence score†	1 (0-2, 273)	1 (0-2, 265)		1 (0-2, 219)	1 (0-2, 255)	0.008		1 (0-1, 218)	1 (0-2, 255)	0.10
Inhaler check in past 6 months	NA	NA		168 (68)	157 (55)	0.006		156 (65)	153 (55)	0.02
Agreed a care plan with healthcare provider‡:										
In past 6 months	80 (28)	82 (29)		108 (44)	85 (30)	0.002		79 (33)	75 (27)	0.02
>6 months ago	25 (9)	26 (9)		53 (21)	70 (25)			72 (30)	70 (25)	
Never	165 (57)	156 (54)		63 (25)	104 (37)			68 (28)	111 (40)	
Written advice on what to do if symptoms get worse	49 (17)	49 (17)		57 (23)	47 (17)	0.05		54 (23)	52 (19)	0.14
Antibiotic rescue pack:										
Has one	77 (27)	75 (26)		93 (37)	81 (29)	0.02		97 (40)	83 (30)	0.02
Confident in its use	71 (92)	68 (91)		85 (91)	76 (94)	0.49		95 (98)	76 (92)	0.09
Steroid rescue pack:										
Has one	56 (19)	51 (18)		63 (25)	60 (21)	0.23		70 (29)	58 (21)	0.03
Confident in its use	52 (93)	47 (92)		53 (84)	55 (92)	0.40		65 (93)	54 (93)	1.00

Percentages do not add up to 100% owing to missing data.

NA=not applicable; IQR=interquartile range.

* Denominator is participants who made a quit attempt in previous 6 months.

† Ranges from 0 to 24. Low scores are good and high scores are bad.

‡ Baseline assessment only covered the previous 12 months (rather than 6 months at follow-up points).

### Sensitivity and subgroup analyses

There were no differences in the findings for the analysis in accordance with the protocol, when regression imputation was used to impute missing data, or when the analysis excluded questionnaires returned either early or late (see web appendix 3). Subgroup analyses also found no evidence that the effect size differed by age, sex, ethnicity, comorbidities, baseline MRC level, smoking status, Hospital Anxiety and Depression Scale, physical activity, predicted forced expiratory volume in one second (≥80 or <80), degree of limitation of activities in the SGRQ-C, active engagers, or self efficacy. There was some evidence of an interaction with baseline SGRQ-C (P=0.04); with a greater benefit for intervention in participants with a baseline score≥25 (ie, those with poorer quality of life) (mean difference −3.0, 95% confidence interval −6.4 to 0.3) compared with those with a baseline score<25 (2.3, −1.6 to 6.2).

### Adverse events

There were 44 serious adverse events reported by participants; 24 in the telephone health coaching arm and 20 in the usual care arm. Five deaths occurred in the intervention group due to cor pulmonale, stroke, ruptured aortic aneurysm, and malignancy (2). None were considered to be related to the intervention.

## Discussion

### Principal findings

This trial is the first to evaluate the effectiveness of a telephone health coaching intervention delivered by a nurse to support self management for patients with mild symptoms of chronic obstructive pulmonary disease (COPD). We showed an improvement in self reported self management activities in the telephone health coaching group compared with usual care, but we did not observe a difference in our primary outcome of health related quality of life measured by the St George's Respiratory Questionnaire (SGRQ-C) (nor in the impact, symptom, or activity domains) although the confidence intervals did include the minimal clinically important difference of four points for the activity and symptom domains. Self reported physical activity was higher at six months in the intervention group, but this was not sustained at 12 months. In addition, activities targeted by the intervention, including patients asking a health care professional to check their inhaler use technique, asking their doctor to agree a care plan, and having a rescue pack were higher in the intervention group at six and 12 months follow-up, compared with usual care. This suggested that a proportion of intervention participants adopted active self management.

### Comparison with other studies

Our approach was new in comparison to other trials of self management and telephone coaching interventions by targeting patients with mildly symptomatic disease. Most previous trials of COPD self management have excluded participants with Global Initiative for Obstructive Lung Disease (GOLD) stage I (mild airflow obstruction),^9^ ^40^ whereas a third of our patients, were in this category, as we particularly wanted to evaluate an intervention for patients with mild COPD, who are a clinically important, but largely neglected group despite having a reduced health related quality of life.^41^

Systematic reviews of self management interventions have reported improvements in COPD related quality of life, measured by the SGRQ with a mean difference of −2.40 at 12 months follow-up.^9^ ^40^ ^42^ All reviews reported effects larger than the −1.3 points difference at 12 months found in our trial. Health related quality of life has been favoured as the main outcome for trials of COPD self management as functional status is important to patients and is sensitive to change, while lung function has a natural variation making it difficult to interpret change over short follow-up periods. Compared with other studies of self management in COPD, even those in milder populations,^39^ the SGRQ-C total score in our study was very low at baseline (representing a good health related quality of life). This potentially led to a floor effect, where change may be unlikely to be achievable, or improvement may only be observed over a much longer period. However, for the activity subscore of the SGRQ-C, the mean difference at 12 months (−3.2) found in our trial compares well with those of the systematic reviews, which report statistically significant mean differences of −2.75 and −2.21.^9^ ^40^ These findings are consistent with the differences found at six months (at the end of the patient self management COPD intervention) in self reported physical activity (International Physical Activity Questionnaire). However, this result of reduced limitations to physical activities was not reflected in the self reported quantity or intensity of physical activity (International Physical Activity Questionnaire) or our objective measures of physical activity where there were no differences between groups at 12 months.

An Australian randomised controlled trial (RCT) of a 12 month intensive telephone health mentoring intervention for patients with moderate to severe COPD in primary care also did not report a difference in the SGRQ at 12 months; but did achieve greater improvements in self management capacity and in COPD knowledge than usual care.^43^ Similar to our trial, a 12 week RCT of an intensive automated telecoaching programme reported improvements in physical activity and the functional domain of the Clinical COPD Questionnaire, but not health related quality of life (COPD Assessment Test) at the end of the 12 week intervention period.^44^ Conversely, an RCT of telephone mentoring for home based walking showed no benefit in exercise capacity in patients with COPD before commencing a pulmonary rehabilitation programme, and also had a high withdrawal rate.^45^ In keeping with our findings, a rapid review of 30 RCTs of the effects of telephone health coaching to support self management of long term conditions reported improvements in health behaviours, but did not find conclusive evidence of improvements in quality of life.^5^

Observed short term improvements in self reported physical activity may have required a longer duration of support or intermittent maintenance activities to sustain changes. Primary care consultations were also lower in the intervention group at six months, which again may reflect the increased telephone contact in this period. A consistent message of the telephone health coaching was for intervention participants to use their routine appointments with primary care for their inhaler use technique to be checked or to discuss a care or action plan and it appears that participants heeded this message and did not book additional consultations for self management advice or support.

### Strengths and limitations of this study

There were many strengths of this study. Firstly, focusing on a mildly symptomatic patient group who are largely excluded from other trials provided novelty and potential for clinical benefit. We used a multicentre study design incorporating a large sample of practices representative of the general UK population; a pragmatic design to accommodate a broad patient group with no selection by motivation to change health behaviours; spirometry was undertaken using trained staff and quality assured and we achieved a good follow-up rate. The intervention was underpinned by social cognitive theory and included techniques such as goal setting that have been shown to be effective in modifying behaviour and was at an intensity that might potentially be delivered in a publicly funded health service.^46^ We achieved good fidelity of delivery of the intervention, with 75% of intervention participants receiving all four calls and only four patients receiving none. There did not appear to be any contamination or change in behaviour in the usual care group with their self reported self management behaviours remaining static throughout the trial. In keeping with the pragmatic nature of this intervention, we did not check whether those who checked inhaler use technique had adequate training, but this is a core component of primary care management of COPD.^24^

Our study has some limitations. The intervention was in a group of people with only mild symptoms of breathlessness, who may not have considered themselves ill, thus a high degree of motivation may have been needed to take part. Highly motivated patients would be more likely to self manage their condition and change lifestyle behaviours. Our sample reported high levels of regular physical activity exceeding the lower recommended amount of moderate or vigorous activity per week at baseline; so, despite our efforts to recruit all eligible patients from primary care there is likely to have been self selection of people to the study, which may have affected capacity to improve, which is a feature of most behaviour change trials. The intervention did not meet the needs of some patients who withdrew from the intervention and in some cases also withdrew from the trial, resulting in an imbalance in follow-up rates between study arms. The patients who withdrew gave reasons including feeling that the intervention did not meet their needs as they were already physically active and some that were too unwell after an exacerbation. This may point to the need for more individual tailoring than actually occurred. In addition, delivery by telephone may give less opportunity for the building of rapport between the patient and nurse. Issues of rapport, acceptability, and tailoring of the intervention will be addressed in more detail in a separate publication of the qualitative evaluation. We did not observe large differences in the characteristics of those who withdrew from the trial, nor any differences in the interpretation of the primary outcome with a sensitivity analysis to assess the impact of missing data. The power calculation was based on detecting a four point difference in the SGRQ-C at 12 months (mean score 39, SD 15). Although participants in patient self management COPD had less severe disease at baseline (mean score 28.7) than expected, the standard deviation was 14.5 meaning we still have 80% power to detect the four point difference. However, owing to the lower SGRQ-C score at baseline, this four point difference now corresponds to a 14% proportional reduction compared with the 10% proportional reduction in the original sample size.

We trained the nurses for two days with further role plays and group calls to discuss challenges once the intervention had started. Our evaluation of the logs of their telephone calls and recorded calls identifies a variation in communication style from a patient centred to a more directive approach. Further, owing to the nature of recruitment across different sites, the distribution of calls completed by nurses was uneven. It was apparent that some patients were reluctant to set physical activity goals. It is possible that longer nurse training would have led to greater communication skills and more behavioural change, but this was a pragmatic study that aimed to evaluate an intervention that could be rolled out in practice. It is possible that a longer intervention duration, with calls beyond three months, would have led to greater effects and that in our group with predominantly mild disease, follow-up beyond 12 months might be needed to detect changes.

### Implications for clinicians and policy makers

Adding telephone health coaching to support self management did not improve health related quality of life in our patient population with only mildly symptomatic disease and who were already quite physically active at baseline. It did, however, lead to an increase in self reported physical activity at six months, which is likely to result in health benefits,^15^ ^16^ and self management activities which are likely to reduce the frequency and severity of exacerbations. While there is still uncertainty about best practice for managing people with mildly symptomatic disease, inhaled therapies are widely used in this group and improved engagement with education about correct delivery technique will help to realise improved outcomes for these patients.^18^ ^19^ Self management support is currently recommended, but it is not likely to be well implemented.^47^ Much evidence for COPD self management support comes from patients recruited from secondary care and there needs to be a synthesis of the findings of support for self management in patients recruited from a primary care setting. It may be that among people with mildly symptomatic disease, self management support should be provided for those with poorest health related quality of life, which is the greatest predictor of future quality of life,^48^ or in those with the most frequent exacerbations.^14^ It may also be that a different health related quality of life outcome measure is needed for people with mild or early COPD that addresses limitations specific to the stage of their disease.

There is a lack of evidence of effective interventions for patients with mild COPD and this trial, while improving some self management behaviours, did not show evidence of clinical benefit. There remains a need to identify successful interventions for patients with milder symptoms of COPD and this also has clear implications for screening or case finding activities, which would identify patients with mild disease, and cannot be recommended while there is a lack of effective treatment options for this patient group. There are wider implications in the use of telephone health coaching; a rapid review reported that it appears to be most effective in vulnerable populations, who have difficulty accessing health services,^4^ which is not reflective of our study population. Supporting self management in patients with early disease, or risk factors, remains a challenge. Apart from diabetes prevention programmes, health services generally focus self management support and rehabilitation services on people with more advanced disease, but there is the potential for considerable health and health service gains if we could facilitate self management in patients with early disease and slow their decline. Establishing whether this is possible will require long term follow-up studies.

### Conclusions and policy implications

A novel telephone health coaching intervention to promote behaviour change in patients with mild symptoms of dyspnoea in primary care led to changes in self management activities, but did not improve health related quality of life. There remains a clear need to identify risk mitigating interventions that can effectively prevent or delay disease progression in this patient group.

**What is already known on this topic**

Current policy for the prevention and management of long term conditions focusses on efforts to prevent onset or slow progression of disease early in the disease trajectory
This prevention paradigm has only recently been adopted for chronic obstructive pulmonary disease (COPD)
Systematic reviews have shown that self management support for patients with COPD is effective in improving health related quality of life and in reducing hospital admissions, but the evidence comes largely from patients with moderate or severe disease and predominantly recruited from secondary care

**What this study adds**

Telephone health coaching comprising components that were theoretically associated with slowing decline of lung function, did improve self management activities that were targeted by the intervention in patients with mildly symptomatic COPD recruited from primary care
Health related quality of life was not improved over the 12 month follow-up period

## Web Extra

Extra material supplied by the author

**Supplementary materials:** Outline of the intervention components

**Supplementary materials:** Accelerometry methods and adherence

**Supplementary materials:** Results of sensitivity analyses for the PSM-COPD study

## Notes

Contributors: KJ led the design of the trial, with contributions and advice from PAC, AD, RJ, CH, SS, PA, SJ, LD, and DF. KJ, RJ, PA, AD, and DF contributed to decisions on outcome measures. DF advised on involving practices. RJ and PA advised on lung function testing. AD and KJ designed the intervention. SJ designed the economic evaluation. KJ, PAC, CH, and Rhian Hughes (Keele University) were the local PIs. CAH and NI developed the statistical analysis plan and undertook the analyses. KJ drafted the paper with MSS, with critical input from all other authors. KJ and MSS contributed equally to this paper. All authors have read and approved the final draft. KJ is the guarantor.

Ethical approval: The study received ethical and research governance approval from the NHS Research Ethics Service, West Midlands, Solihull (Reference 13/WM/0206). All participants gave informed consent.

Data sharing: Data from the patient self management COPD study or the interventional materials are available from the corresponding author at c.b.jolly@bham.ac.uk. All the individual participant data collected during the trial (including the data dictionary) will be available, after deidentification, immediately after publication with no end date. The study protocol has been published. All proposals requesting data access will need to specify how it is planned to use the data, and all proposals will need approval of the trial co-investigator team before data release.

Transparency: The guarantor (KJ) affirms that the manuscript is an honest, accurate, and transparent account of the study being reported; that no important aspects of the study have been omitted; and that any discrepancies from the study as planned (and, if relevant, registered) have been explained.
