# Supplementary material for: Self management of patients with mild COPD in primary care: randomised controlled trial
Source: BMJ. 2018 Jun 13;361:k2241. doi: 10.1136/bmj.k2241 (PMC5998171; doi:10.1136/bmj.k2241)
Supplement: Supplementary file 1 — Supplementary materials: Outline of the intervention components [file jolk042829.ww1.pdf]

Web appendix 1: Outline of the intervention components

Week 1

Telephone:

Medication management: Inhaler technique: timing of last check, set goal to get technique checked. Medication adherence (if relevant) –memory prompts, restructuring environment, support from partner.

Smoking: Discussion of smoking behaviours, quit attempts and barriers to quit. Encouragement to set goal to contact smoking cessation service and to seek social support to support a quit attempt.

Physical activity: current levels and breathlessness, goal to increase activity (duration and or intensity) and record in diary.

Action planning: discussion of management of exacerbations, did they have written action plan, confidence with use of rescue pack. Prompt to discuss at routine appointment with GP if lacking understanding or confidence.

Postal:

Physical activity booklet including information on benefits for COPD and overcoming barriers

Physical activity diary

Pedometer with instructions

Smoking information booklet (smokers only) with contact details of smoking cessation service

Inhaler technique instruction leaflet

↓

Week 3

Telephone:

Discussion of progress with goals set in previous session and any barriers to achieving goals.

Review of physical activity levels and setting of new goals.

Discussion of smoking, medication management and action planning as required.

Postal:

Information on opportunities for physical activity in the locality

Information leaflet: What are SMART goals?

SMART goals sheet

↓

Week 7

Telephone:

Discussion of progress with goals set in previous session and any barriers to achieving goals.

Review of physical activity levels and setting of new goals.

Discussion of smoking, medication management and action planning as required.

Postal:

SMART goals sheet

↓

Week 11

Telephone:

Discussion of progress with goals set in previous session and any barriers to achieving goals.

Review of physical activity levels and setting of new goals.

Discussion of smoking, medication management and action planning as required.

Postal:

SMART goals sheet

↓

Week 16

Postal SMART goals sheet

↓

Week 24

Postal:

Information on opportunities for physical activity in the locality

Leaflet on tips for sustaining physical activity
