# Supplementary material for: Self management of patients with mild COPD in primary care: randomised controlled trial
Source: BMJ. 2018 Jun 13;361:k2241. doi: 10.1136/bmj.k2241 (PMC5998171; doi:10.1136/bmj.k2241)
Supplement: Supplementary file 2 — Supplementary materials: Accelerometry methods and adherence [file jolk042829.ww2.pdf]

Web appendix 2: Accelerometry methods and adherence

At the baseline assessment a GENEactiv accelerometer was fitted on the participant's non-dominant wrist, which they were asked to return by post in a pre-paid envelope after 7 days of continuous wear. At 12 month follow-up accelerometers were posted to participants with a follow-up telephone call to explain how to start the recording. They were asked to wear them continuously for 7 days and return them by post. Non-responders were telephoned and given the option of completing the questionnaire over the telephone.

Analysis

Data were collected in 60 second epochs. In order to be considered a valid day, participants needed to wear the accelerometer for at least 10 waking hours. A minimum of 5 days of valid data was necessary for inclusion in the final analysis. The criterion for non-wear was 120 minutes of zero counts. The default cut-points for adult physical activity were used to determine the amount of time spent in moderate to vigorous physical activity. These cut-points in g·min were 483 for the threshold from sedentary to light activity, 678 for moderately intense activity and 2264 for vigorous activity.

Adherence

At baseline 535 participants returned an accelerometer with data on, of which 522 (90.5%) had valid data for inclusion in the analyses. At 12 months follow-up, 416 accelerometers were returned with data, of which 411 had valid data for analysis; this was 71.2% of the initial sample.
