# Supplementary material for: Self management of patients with mild COPD in primary care: randomised controlled trial
Source: BMJ. 2018 Jun 13;361:k2241. doi: 10.1136/bmj.k2241 (PMC5998171; doi:10.1136/bmj.k2241)
Supplement: Supplementary file 3 — Supplementary materials: Results of sensitivity analyses for the PSM-COPD study [file jolk042829.ww3.pdf]

Web appendix 3: Results of sensitivity analyses for the PSM-COPD study

	Baseline		12 months			
	**Telephone PSM** mean (sd)	**Usual Care** mean (sd)	**Telephone PSM** mean (sd)	**Usual Care** mean (sd)	**Mean difference[1] (95% CI)**	**p-value**
**Per-protocol analysis**						
SGRQ-C total	26.5 (13.5)	29.5 (14.5)	27.6 (14.6)	30.8 (17.0)	-1.4 (-3.6, 0.8)	**0.2**
SGRQ-C symptom	48.1 (21.1)	47.8 (20.7)	49.2 (21.2)	50.0 (22.7)	-1.9 (-4.9, 1.2)	**0.2**
SGRQ-C activity	34.1 (19.8)	38.7 (21.3)	33.9 (20.1)	39.3 (24.4)	-2.4 (-5.6, 0.8)	**0.1**
SGRQ-C impact	14.4 (12.3)	17.5 (13.9)	15.8 (13.8)	19.3 (15.6)	-1.6 (-3.8, 0.5)	**0.1**
**Analysis to assess impact of assessment window**						
SGRQ-C total	27.1 (13.9)	29.1 (14.2)	28.1 (15.5)	30.7 (16.8)	-1.1 (-3.3, 1.1)	**0.3**
SGRQ-C symptom	48.6 (21.2)	47.7 (20.7)	49.3 (21.4)	50.0 (22.6)	-1.6 (-4.7, 1.4)	**0.3**
SGRQ-C activity	35.0 (19.9)	38.5 (21.1)	34.4 (20.7)	39.1 (24.2)	-2.5 (-5.6, 0.7)	**0.1**
SGRQ-C impact	14.8 (12.8)	17.0 (13.4)	16.4 (14.8)	19.2 (15.3)	-1.1 (-3.3, 1.0)	**0.3**
**Imputation to assess impact of missing data**						
SGRQ-C total	27.9 (14.5)	29.5 (14.5)	28.5 (15.9)	31.1 (17.3)	-1.4 (-3.4, 0.6)	**0.2**
SGRQ-C symptom	48.3 (21.8)	47.7 (20.5)	48.6 (21.1)	50.2 (22.4)	-2.0 (-4.7, 0.7)	**0.1**
SGRQ-C activity	36.3 (21.0)	38.5 (21.2)	34.7 (21.4)	39.2 (23.8)	-3.0 (-5.9, -0.2)	**0.04**
SGRQ-C impact	15.5 (13.4)	17.6 (13.8)	17.5 (15.3)	19.9 (15.5)	-0.9 (-2.9, 1.1)	**0.4**

[1]Telephone PSM compared to usual care (negative values favour Telephone PSM).
